# Pleomorphic adenocarcinoma of the lacrimal gland with multiple intracranial and spinal metastases

**DOI:** 10.1186/1477-7819-5-29

**Published:** 2007-03-07

**Authors:** Jung Yong Ahn, Jong Hee Chang, Se Hoon Kim, Kyu Sung Lee

**Affiliations:** 1 Department of Neurosurgery, Yonsei University College of Medicine, Seoul, Republic of Korea; 2 Department of Pathology, Yonsei University College of Medicine, Seoul, Republic of Korea

## Abstract

**Background:**

Pleomorphic adenoma of the lacrimal gland is known to undergo malignant transformation when incompletely excised. Even if such a malignant change occurs, intracranial direct invasion and leptomeningeal seeding are seldom encountered.

**Case presentation:**

A 50-year-old woman presented with malignant transformation associated with both intracranial invasion and multiple intracranial and spinal disseminations in the third recurrence of pleomorphic adenoma of the lacrimal gland, 6 years after initial treatment. MRI demonstrated increased extent of orbital mass, extending to the cavernous sinus. The patient underwent intensity-modulated radiation therapy (IMRT) and Gamma Knife radiosurgery. Follow-up MRI showed multiple leptomeningeal disseminations to the intracranium and spine.

**Conclusion:**

It is important to recognize that leptomeningeal intracranial and spinal disseminations of pleomorphic adenocarcinoma can occur, although it is extremely rare. To our knowledge, we report the first case of pleomorphic adenocarcinoma of the lacrimal gland presumably metastasizing to the intracranium and spine.

## Background

Pleomorphic adenomas are the most common benign epithelial tumors affecting the lacrimal gland [1]; however, their malignant counterpart, the malignant mixed tumor, is much less common [2]. Pleomorphic adenoma is known to recur and also to undergo malignant transformation when incompletely excised [3,4].

Even if such a malignant change occurs, intracranial direct invasion and intracerebral and spinal metastases are seldom encountered, because the tumor itself is well demarcated [5]. We present a case of malignant transformation associated with both intracranial invasion and multiple intracranial and spinal disseminations in the third recurrence of pleomorphic adenoma of the lacrimal gland, 6 years after initial treatment. We also describe an initial experience with gamma knife radiosurgery for intracranial invasion. To the best of our knowledge, this is the first report of pleomorphic adenocarcinoma with presumed multiple intracranial and spinal metastases treated with Gamma Knife radiosurgery.

## Case presentation

**Figure 1:**
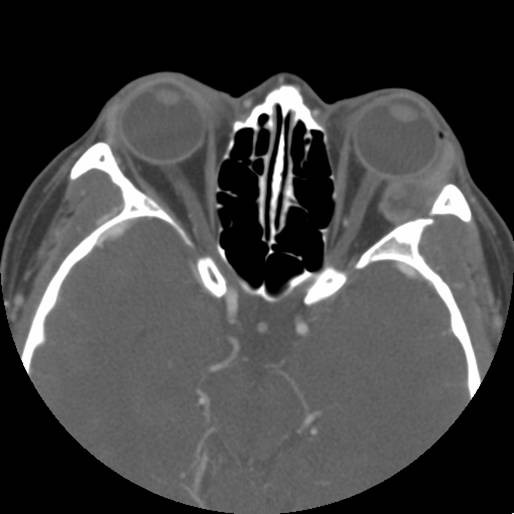
Contrast-enhanced axial orbital computed tomography showing a well-circumscirbed mass at lateral aspect of the left orbit without bone involvement.

**Figure 2:**
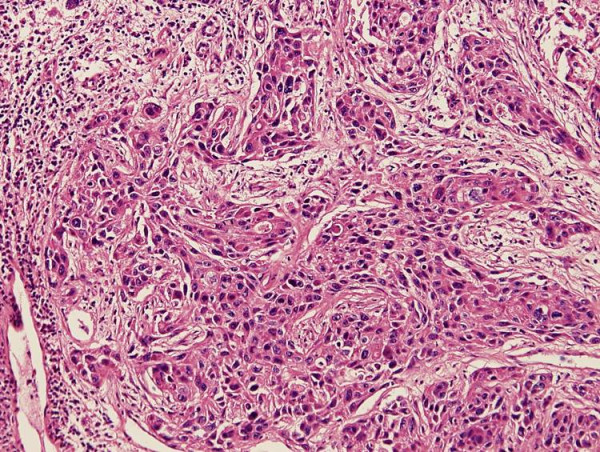
Hematoxylin-eosin staining (100× magnification) showing poorly differentiated adenocarcinoma, suggesting malignant carcinomatous changes in the recurrent pleomorphic adenoma. Nuclear atypia and prominent mitosis are noted.

A 50-year-old woman had lacrimal mass presented with progressive exophthalmos and visual disturbance in the left eye in 2000. Orbital CT scan showed a well-circumscirbed mass at lateral aspect of the left orbit without bone involvement (Figure 1). She underwent subtotal resection of a smooth, encapsulated, and multilobulated mass at the referring hospital. The histological diagnosis was pleomorphic adenoma of the lacrimal gland. The clinical course was uneventful until 4 years after the operation, when a local recurrence with protrusion of the left eye and swelling of the left temporal muscle area required subtotal resection at the Department of Ophthalmology, Yonsei University Hospital. The mass was extended to periorbital tissue, lateral orbital rim, and temporal muscle. The histological diagnosis was poorly differentiated adenocarcinoma, suggesting malignant carcinomatous changes in the recurrent pleomorphic adenoma (Figure 2). MRI surveillance of the lesion was initiated. Six months after second surgery, MRI demonstrated increased extent of orbital mass, extending to the cavernous sinus. The patient underwent intensity-modulated radiation therapy (IMRT) with two different planning target volumes (PTVs): 1) 45 Gy to extracranial portion, 2) 30 Gy to intracranial portion.

**Figure 3:**
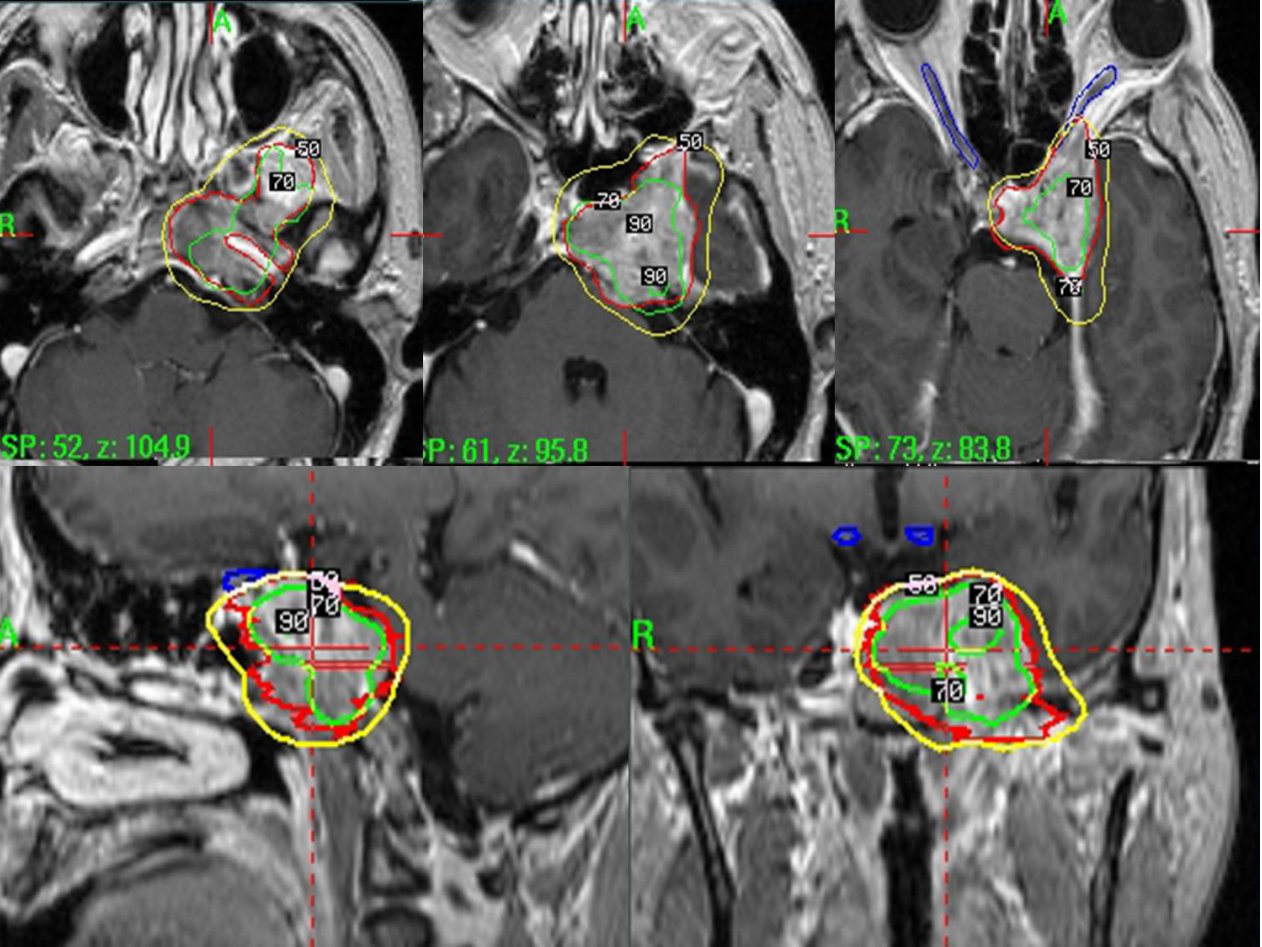
Showing the treatment plan of gamma knife radiosurgey.

Six month later, the patient complained of the left eye pain and the left facial paresthesia. An ophthalmological examination revealed ptosis, exophthalmos, and ophthamoplegia of the left eye. Facial sensory change on the left whole trigeminal territories was noted. MRI revealed a well-circumscribed mass of the left cavernous sinus extending to the infratemporal fossa and clivus. The patient underwent Gamma Knife stereotactic radiosurgery with a marginal dose of 15 Gy and maximum dose of 30 Gy to 29.7 ml of tumor volume. Dose was adjusted to the right optic nerve and optic chiasm (8.4 Gy) and to the left optic nerve (15 Gy) (Figure 3). Intentional high dose to the left optic nerve was planned due to useless vision caused by the left complete occulomtor nerve palsy.

**Figure 4:**
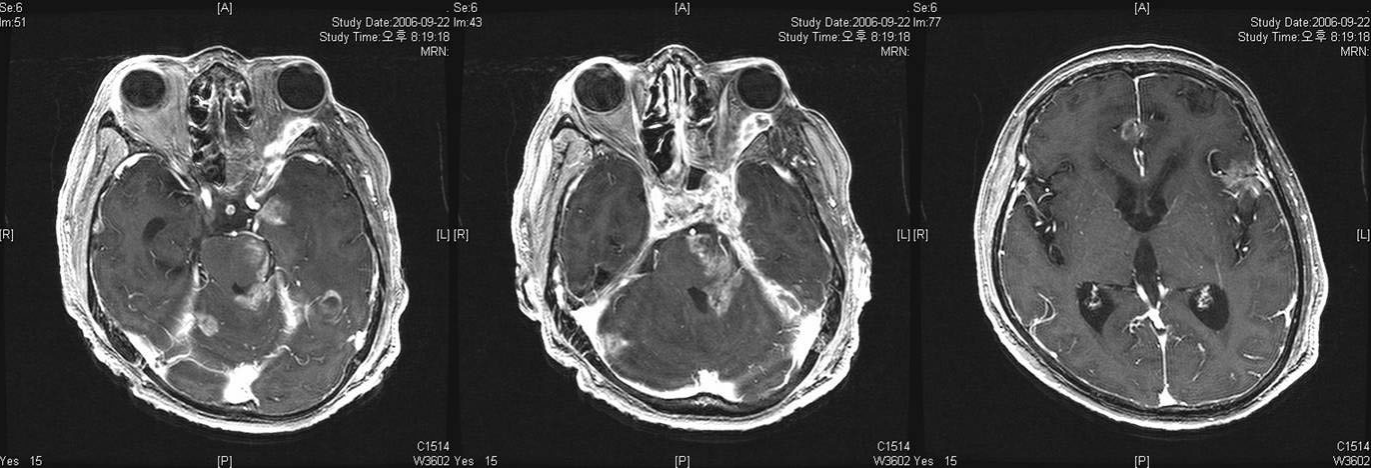
Follow-up brain MR images revealing a slightly decreased tumor size extending to orbital, cavernous sinus, infratemporal fossa, and clivus. However, small nodular enhancing lesion on the interhemispheric fissue and superior cerebellar peduncle, and leptomeningeal seeding is suspected.

**Figure 5:**
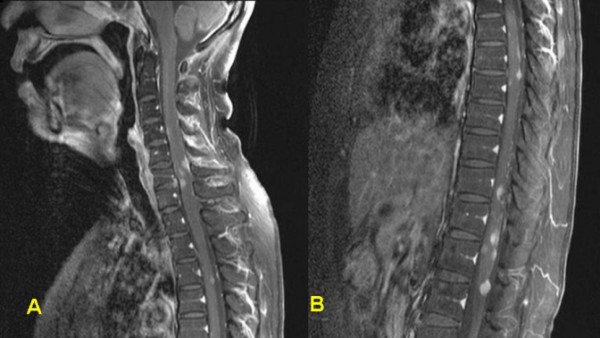
Spinal sagittal MR images (A: cervical, B: thoracic) revealing multiple nodular enhancing masses on the cervico-thoracic area.

Six months later, follow-up MRI revealed a slightly decreased tumor size extending to orbital, cavernous sinus, infratemporal fossa, and clivus. However, small nodular enhancing lesion on the interhemispheric fissue and superior cerebellar peduncle, and leptomeningeal seeding was suspected (Figure 4). Whole spinal MRI was indicated due to neck and back pain, and it revealed multiple nodular enhancing masses on the cervico-thoracic area (Figure 5). Palliative radiotherapy for the intracranial and spinal metastases was planned, but the patient refused further active treatment. The patient was discharged and underwent hospice care. Five months later, the patient died due to pneumonia and sepsis related to terminal stage of metastatic cancer.

## Discussion

Pleormorphic adenoma is the most common benign epithelial lacrimal gland tumor. The incidence of pleomorphic adenoma in the literature has been reported as 9–27% of lacrimal gland tumor [1,3]. Pleomorphic adenoma presents an excellent prognosis, particularly following complete excision with the pseudocapsule intact [6]. If the tumor is incompletely removed, recurrence may occur, usually taking many years to become clinically apparent. Although recurrent tumors are usually as benign as the original, some become frankly malignant many years (mean 17.4) after the initial excision of a pleomorphic adenoma [6-11]. The malignant components of pleomorphic adenoma are usually carcinomatous, showing features of either an adenocarcinoma or a poorly differentiated carcinoma, sometimes sarcomatous. Furthermore, malignant evolution in a tumor recurrence may be associated with intracranial invasion to the cavernous sinus, although most recurrences involved only intraorbital structures. Intracranial infiltration following malignant transformation in recurrent lacrimal gland tumors have previously been reported [7-10]. In our case, pleomorphic adenoma was evolved into both intracranial invasion and presumed intracranial and spinal disseminations in the third recurrence of pleomorphic adenoma of the lacrimal gland.

The recommended treatment for suspected pleomorphic adenoma of the lacrimal gland is complete surgical resection or en block excision. However, incomplete or piecemeal removal of the lesion or rupture of its pseudocapsule inevitably results in a recurrence, which may evolve malignant changes in the original tumor [8,10]. A biopsy of the lesion may cause the seeding of tumor cells into the orbit and a clinical recurrence [12]. Thus, it is emphasized that complete excision of the lesion within the pseudocapsule is essential to prevent the potential malignant evolution in recurrent pleomorphic adenomas [10,11].

The optimal treatment of the malignant mixed tumors remains controversial. An aggressive approach, such as orbital exenteration followed by local radiotherapy, is recommended by some authors [2,13]. However, Polito and Leccisotti [14] did not find improved survival in patients submitted to extensive surgery, when compared with those that underwent more limited local extension. Radiation therapy may be an adjuvant in the management of extensive or recurrent pleomorphic adenomas even following complete excision. On the other hand, it could be argued that radiation therapy is of little value as a salvage procedure for recurrent or residual malignant mixed tumors [15]. Although irradiation can provide short-term palliation, it achieves little in terms of long-term tumor control. Chemotherapy may occasionally be clinically efficacious for the recurrence of pleomorphic adenoma [10,11]. In present case, local control was achieved by IMRT and gamma knife radiosurgery, but leptomeningeal seedings were encountered even though local control was more or less successful.

The malignant tumors following an intermediate course and are associated with a median survival of 12 years and a 15-year survival rate of 46% [5,11,16]. In contrast, patients with tumor recurrence particularly associated with intracranial invasion following malignant changes have a median survival of less than 5 years [7-9]. This patient died 17 months after the first intracranial extension and 6 years after the first operation.

## Conclusion

To our knowledge, we report the first case of pleomorphic adenocarcinoma of the lacrimal gland presumably metastasizing to the intracranium and spine. Our initial experience with intensity-modulated radiation therapy and Gamma Knife radiosurgery for intracranial direct invasion of the pleomorphic adenocarcinoma was of little value for as a salvage procedure for malignant transformation of pleomorphic adenoma. This case emphasize that complete excision of the original lesion is essential to prevent the potential malignant evolution in recurrent pleomorphic adenomas.

## Competing interests

The author(s) declare that they have no competing interests.

## Authors' contributions

**JYA **conceptualized the study, gathered the data and drafted the manuscript. **JHC **participated in Gamma Knife radiosurgery and took charge of post-operative managements together with JYA. **SHK **carried out the pathologic studies. **KSL **supervised the process and finally approved the manuscript for been published. All authors have read and approved the final manuscript.
